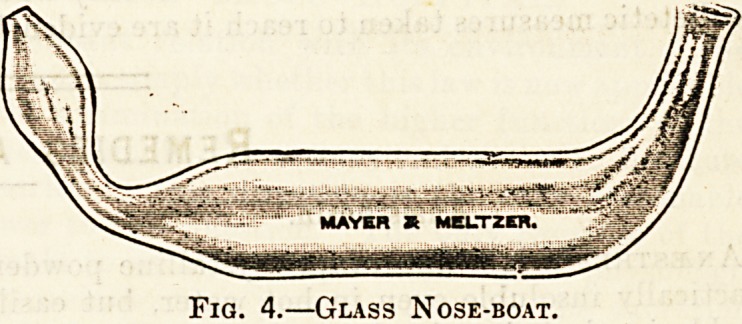# Irrigants and Syringes: Their Defects and Dangers

**Published:** 1906-10-06

**Authors:** Wyatt Wingrave

**Affiliations:** Central London Throat and Ear Hospital


### New Surgical Methods.

### IRRIGANTS AND SYRINGES: THEIR DEFECTS AND DANGERS.

By Wyatt Wingrave, M.D., Central London Throat and Ear Hospital.

### Objects.

The object of a douche or irrigant is twofold: first to remove morbid secretions, accumulations, and foreign substances; secondly either to kill, retard, or prevent the growth of bacteria. Since fœtor is generally the result of bacterial changes, its correction generally coincides with antisepsis. We will discuss the simplest means of attaining these objects, chemically and mechanically.

### Essentials of Irrigant.

In the selection of material and method we shall obviously be influenced by the nature of the discharge and its source. There are, however, certain essentials of an irrigating solution which must first be considered: (1) The solution should, wherever practicable, be a solvent of the substance to be removed. (2) The material should be readily soluble or miscible with water. (3) When used for sensitive surfaces and mucous membranes it should be non-irritating. (4) It should possess the power of penetrating the surface tissues to ensure thoroughness. (5) It should be a real antiseptic, and not merely a deodorant. (6) It should be economical in cost and non-poisonous. Of these essentials perhaps one of the most important is the first, because so many irrigants are used which either have no such power or even actually precipitate and harden the substances which they are employed to remove.

### Nature of Discharges.

The next consideration must be a knowledge of the nature of the substance to be removed.

*Catarrhal* products vary much in consistence: they may be thin, serous, viscid, or even dry and encrusted. They for the most part contain mucin, soluble in alkalies; cell-globulin, and albumen, soluble in neutral salts such as sodium sulphate and chloride.

*Pus* also varies according to its age and source; it is soluble in sodium sulphate and most neutral salts.

*Wax*, or *Cerumen* may be soft or hard. When soft it is readily removed by weak solution of bicarbonate of soda mixed with a mild antiseptic such as lysoform; but when hard is generally mixed with hairs and epithelial scales, which should be first softened with an alkali in glycerin before syringing (sodium hydrate 1 per cent.).

All incrustations and dried secretions should first be softened with an alkaline solution such as borax or sodium bicarbonate (1 per cent. to 5 per cent.). Membranous exudations, as in diphtheria, are best removed by solutions of sodium sulphate or lime water; the same remark applies to blood-clots.

### Antiseptics.

We will now consider the antiseptics—a somewhat difficult matter, because although there are so many, yet there are few which can be relied upon, and none which can be used indiscriminately. A good plan is to classify them into poisonous and non-poisonous; but in doing so only those which experience has proved to be really practicable will be referred to. The chief poisonous ones are biniodide and perchloride of mercury. Their properties are so well known that discussion is superfluous. Zinc permanaganate is safe if used in very weak solution. Carbolic acid is popular, but has many drawbacks. It is very poisonous; it temporarily paralyses the senses of smell and taste and touch; its solution is difficult to prepare, is very irritating to all mucous membranes, consequently should only be employed with the greatest care, and in very weak solutions to the nose, ear, and eyes. It is often merely suspended in the water, not dissolved, therefore is better prepared from its glycerin solution, and combined with borax or bicarbonate of soda when employed on mucous membranes. Formalin, although non-poisonous in weak solutions, may be used for ordinary wounds if very weak, but *never* to the nose or throat because of its irritating pungency. Of the non-poisonous, or at all events those which are non-toxic in the strength as employed, are: (1) Lysoform, (2) sanitas, (3) potass. permanganate, (4) chinosol, (5) hydrogen peroxide, (6) creolene (medical), (7) cyllin, (8) liq. carbonis detergens (9) izal. The most reliable of all these are cyllin, lysoform, and sanitas for general use. Lysoform owes its antiseptic power to formic aldehyde, but more thoroughly cleanses, being incorporated with soap. Unfortunately these only form emulsions with water, and therefore are opaque. Hydrogen peroxide must be used with care in any cavity because of the brisk effervescence when in contact with pus, blood, etc.

Chinosol is a very safe but expensive drug; it mixes with sod. sulphate, and gives a clear solution.

When there is fœtor cyllin, lysoform, sanitas, creolin, liq. carbonis detergens, izal are all reliable at a strength of 1 per cent. Boric acid is not to be commended, since it is not only a bad solvent of any discharge, but its antiseptic properties are so feeble as to be practically unreliable. The different combinations of eucalyptus, thymol, etc. are at the best but expensive cosmetics and deodorants, and cannot be seriously considered as antiseptics. A solution of 1 per cent. of common salt is always preferable to simply boiled water, as it is a solvent of pus. Sodium sulphate (Glauber's salt) is, however, more preferable; it is not an antiseptic, but its value is greatly due also to its great penetrating power. It is easily combined with biniodide.

### Formulæ.

It will be seen that it is somewhat difficult to select an universal irrigant—*i.e.* one which is suitable for all purposes—but there can be no harm in suggesting the following formulæ as being simple, reliable, and applicable to the purposes indicated.

A simple mechanical or cleansing irrigant for all purposes, including ear and nose:—

| | |
|---|---|
| Sodium sulphate ... ... ... | 2 drachms |
| (The pure exsiccated sulphate should be used.) | |
| Borax or sodium carbonate ... | 40 grains |
| Water ... ... ... ... | to one pint |

Antiseptic douche for nasal or any mucous membrane (non-irritating):—

| | |
|---|---|
| Sodium sulphate ... ... ... | 2 drachms |
| Borax ... ... ... ... | 40 grains |
| Chinosol ... ... ... ... | 10 grains |
| Sanitas ... ... ... ... | 2 teaspoonfuls |
| Water ... ... ... ... | to one pint |

A general irrigant for wounds:—

| | |
|---|---|
| Sodium sulphate ... ... ... | 2 drachms |
| Lysoform ... ... ... ... | 1 drachm |
| Water ... ... ... ... | to one pint |

or

| | |
|---|---|
| Sodium sulphate ... ... ... | 1 drachm |
| Red iodide of mercury ... ... | 2 grains |
| Sodium iodide ... ... ... | 2 grains |
| Water ... ... ... ... | to one pint (1—5000) |

or

| | |
|---|---|
| Sodium sulphate ... ... ... | 1 drachm |
| Perchloride of mercury ... ... | 2 grains (1—5000) |

### Infection by Syringe.

One of the principal objects of this paper is to emphasise the importance of strict cleanliness, and to avoid, not only the conveyance of infection from one wound to another, but to prevent *reinfection*; for, in spite of persistent syringing and irrigation, cases become chronic simply because the faulty act of syringing reinfects the cavity, and bacteria are replanted time after time. Let us take the case of a suppurating ear, sinus or cavity; it is syringed out with an effective antiseptic, say 1 in 1,000, of sublimate solution. The nozzle is placed in the ear or wound, it is emptied, and the cavity is washed out. The contaminated nozzle is then dipped into the irrigating fluid and the syringe is refilled, sucking up at the same time any bacteria which may be sticking to it, so infecting piston and lining. It is either applied to the same wound again or, worse still, to the opposite ear. Such goes on over and over again, and the wound never becomes free from sepsis.

This is not a fanciful sketch, but is one which may be seen any day, and is familiar to every nurse, not only in private, but also in hospital practice.

Such a syringe, even if frequently washed, is not aseptic, and, if examined bacteriologically, will prove to be a perfect zoological garden of germs. The worse offenders in this way are the ordinary india-rubber ball syringe and the glass or metal piston syringe of the drug stores, which, as they can only be filled and emptied by the same aperture, are real death-traps.

No matter how careful one may be in the choice of antiseptics, if this method of syringing be employed asepsis is impossible, and the ordinary apparatus sold at chemists' is a dangerous weapon.

### Choice of Syringe.

The ordinary enema syringe, which is filled at one end and emptied at the other, is a vast improvement; but even this has its drawbacks, since the nozzle becomes infected if the interior remain sterile unless special precautions are taken.

How can this risk of reinfection be avoided? Very easily indeed by always using a detachable nozzle to the syringe. Such as is done in the case of vagina and rectum; therefore why not for the ear, nose, and every other region? If a piston syringe be employed it should be capable of being taken to pieces and thoroughly boiled. The "packing" of the piston should be specially attended to if not of metal. The cotton or worsted should be changed each time it is used, for this is a favourite hiding-place for bacteria.

The Medical Supply Association make a very good aseptic piston syringe (fig. 1) with a detach-

Fig. 1.—Author's Aseptic Syringe. Specially Designed.

able nozzle of various sizes which taken to pieces, can be readily sterilised.

A very simple expedient is to use short lengths of rubber tubing over the nozzle, as in fig. 2, which can be removed before refilling the syringes and afterwards either boiled or thrown away. By this means the nozzle is kept free from active contact with the wound or discharge, so reducing the liability of infecting its interior. Another plan is to use an enema type of syringe with an *inlet* valve, and an *outlet* valve at the nozzle, as designed by the writer and made by Mayer and Meltzer (fig. 2).

Fig. 2.—Author's Aseptic Syringe. Specially Designed for Patient's Own Use as an Aural Douche.

This ensures filling solely by the end *not* brought in contact with the wound, and so prevents contamination of the inside. Bone and ivory nozzles should never be employed, because it is practically impossible to keep them sterile. The hydrostatic douche with a detachable nozzle is preferable when large quantities of fluid are to be employed, but it is very little use in aural work. Messrs Mayer and Meltzer now supply a glass nozzle fitted with an exit valve which can be adjusted to any syringe at a small cost.

Fig. 3 is an excellent syringe designed by Dr. Dundas Grant. It is filled by a lateral tube and tap, which keeps the interior sweet, but requires a supplementary nozzle.

Fig. 3.—Two Way Syringe.

Fig. 4 illustrates the ordinary "nose-boat" or nasal douche, which is fairly safe for self-use; it can be kept clean, and is less liable to do harm than a piston syringe.

In conclusion a caution regarding the indiscriminate and careless use of the nasal douche may not be out of place, for not only is there risk of causing serious ear trouble, but also inflammation of

Fig. 4.—Glass Nose-boat.

the accessory sinuses, impairment of the sense of smell, and chronic nasal disease.

## Figures and Tables

**Fig. 1. f1:**
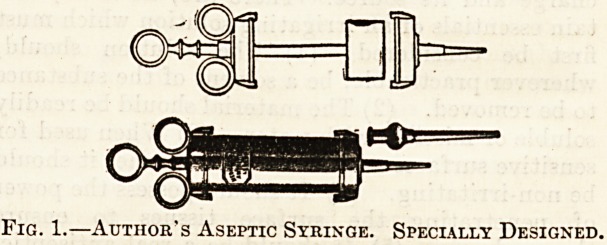


**Fig. 2. f2:**
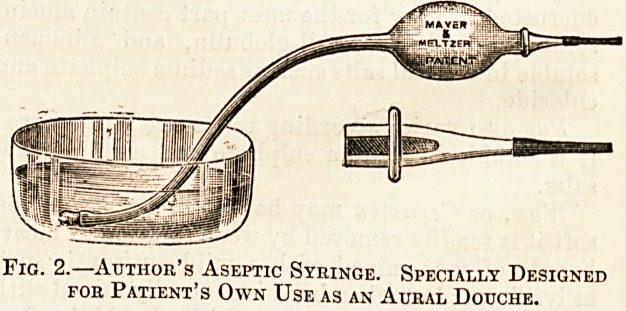


**Fig. 3. f3:**
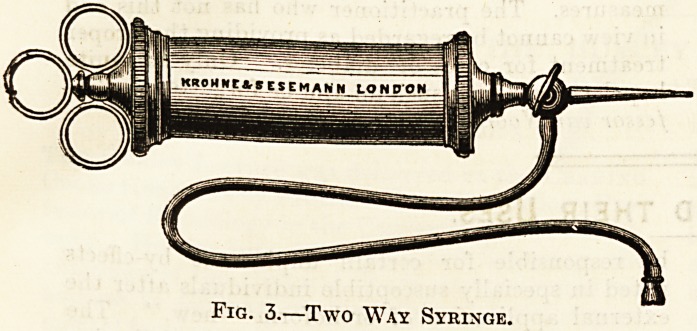


**Fig. 4. f4:**